# Postmenopausal women's usage of complementary and alternative medicine and its relationship to sexual function: A cross‐sectional study in southeastern Iran

**DOI:** 10.1002/hsr2.1470

**Published:** 2023-08-01

**Authors:** Mahlagha Dehghan, Zahra Isari, Mohammad Hossein Abbaszadeh, Seyed Amirreza Shafiee Babaei, Zahra Kamali Narab, Alireza Malakoutikhah, Asma Ghonchehpour

**Affiliations:** ^1^ Department of Critical Care Nursing, Nursing Research Center, Razi Faculty of Nursing and Midwifery Kerman University of Medical Sciences Kerman Iran; ^2^ Nursing Research Center, Razi Faculty of Nursing and Midwifery Kerman University of Medical Sciences Kerman Iran; ^3^ Student Research Committee, Razi Faculty of Nursing and Midwifery Kerman University of Medical Sciences Kerman Iran; ^4^ Nursing Research Center, Razi Faculty of Nursing & Midwifery Kerman University of Medical Sciences Kerman Iran

**Keywords:** alternative medicine, complementary medicine, postmenopause, sexual function

## Abstract

**Background and Aims:**

Sexual dysfunction is one of the most common problems in postmenopausal women that affect their lives. Due to the widespread disadvantages and age limit of drug and hormone therapy, the demand for complementary and alternative medicine (CAM) methods has increased.

**Methods:**

This was a descriptive‐analytical study. A total of 297 postmenopausal women who were referred to health centers in Kerman, southeastern Iran, were selected during the years 2020–2021. The use of CAM and sexual function were examined by Complementary and Alternative Medicine Questionnaire and the Female Sexual Function Index, respectively. Data were collected through a combination of face‐to‐face and online forms.

**Results:**

More than half of the participants used at least one type of CAM in the preceding year for any general reason. The mean satisfaction score for CAM use was 21.7 ± 5.84; Most reasons of use were for improving quality of life, improving physical function, reducing sleep disorders, improving mood status, reducing vasomotor symptom, and improving sexual function, respectively. Most reasons of use were related to sexual dysfunction. Specifically, the mean score for sexual function was 17.96 ± 7.50 which was lower than the scale's midpoint of 28. According to different subscales of sexual function, 52.9% of participants had good desire, 49.5% good arousal, 34.7% good lubrication, 56.9% good orgasm, and 2% had no pain, and 52.5% were satisfied with their sexual function. There were no significant differences between CAM users and nonusers about sexual functions and all its subscales.

**Conclusion:**

There were no significant differences between CAM users and nonusers about sexual functions and all its domains. Further research in different communities with different health systems is recommended to investigate the relationship between CAM and sexual function among postmenopausal women.

## 1 INTRODUCTION

Menopause refers to the permanent cessation of menstruation for a minimum of 12 consecutive months, which occurs due to a lack of estrogen and is not linked to any underlying medical condition.^1^ All these changes can lead to difficulty sleeping, a reduced sex drive (libido), urinary incontinence, palpitations, osteoporosis, recurrent urinary tract infections (UTIs), vaginal dryness and pain,^2^ mood changes, such as low mood or anxiety and depression.^3^ In some societies today, 95% of women enter this stage. This population is projected to reach 1.2 billion by 2030, with an annual increase of 47 million new cases.^4^ In Iran, it is expected that in 2021 there would be about 5 million women of menopausal age due to the aging of the population.^5^

As a woman ages and goes through natural menopause, her sexuality is adversely affected, particularly in terms of libido, arousal, orgasm, desire, and sexual activity.^6^ Sexual dysfunction, one of the most common problems affecting postmenopausal women, significantly impacts their quality of life and can negatively affect self‐esteem, contribute to the development of depression and stress, and impact marital satisfaction.^7^, ^8^, ^9^

The prevalence of sexual dysfunction among women has been reported at up to 63%, which exceeds this rate after menopause and reaches 68%–86%.^9^ This population had increasingly attracted public, pharmaceutical, and medical attention.^10^ To prevent and treat sexual dysfunction and reduce menopausal symptoms during menopause in women, pharmacological methods such as hormonal and nonhormonal methods, complementary and alternative medicine (CAM) are used.^5^, ^6^, ^11^ While hormone replacement therapy is effective in addressing urogenital problems and improving sexual function, concerns about its long‐term use and potential risks, such as cancer and venous thromboembolism, have led women to seek nonpharmacological alternatives.^12^ Therefore, women's concern about the consequences of these treatments has led their tendency to nonpharmacological methods such as CAM, which, in addition to have fewer side effects, is also more cost‐effective.^11^, ^13^, ^14^, ^15^

CAM is a treatment that falls outside of mainstream healthcare that has shown through science to be safe and effective.^16^ Studies have shown varying prevalence rates of CAM use among menopausal women, ranging from 9.8% to 76% in different countries, with Asian countries exhibiting higher figures in recent years.^17^ In Iran, this prevalence is 42%.^18^ Özcan et al. reported that women in menopause used the following methods to overcome insomnia: herbal supplements (96.6%), dietary supplements (98.8%), religious practices (95.7%), and mind–body practices (76.9%).^19^ Research on CAM treatments commonly used for menopausal symptoms indicates that mind–body practices, such as hypnosis, can effectively reduce hot flashes and stress, while the efficacy of natural products remains inconclusive with some safety concerns.^20^ Recent research had reported on the effectiveness of Mediterranean diet, acupuncture, aphrodite, and fennel vaginal cream^18^, ^21^, ^22^, ^23^ on the sexual function of postmenopausal women, however, the findings of Amiri Pebdani et al. suggested that, despite the herb *Ginkgo biloba's* efficacy in increasing sexual desire in women, it had no influence on their sexual activity.^24^

Given the increasing population of postmenopausal women, their common symptoms and complications, and the limitations and potential risks associated with drug and hormone therapies, there is a growing demand for CAM interventions. However, the types of CAM used are influenced by societal, historical, and occasionally religious factors,^25^ Furthermore, there is a lack of sufficient evidence on the use of CAM specifically in Iran. Therefore, it is necessary to investigate the utilization of various CAM methods and their relationship with sexual dysfunction among postmenopausal women. The aim of this study is to examine the use of CAM and its association with sexual function among postmenopausal women attending health centers in Kerman in 2020.

## 2 OBJECTIVES OF THE STUDY

1. What are the patterns of use of different types of CAM among postmenopausal women?
2. What are the reasons for the use of different types of CAM among postmenopausal women?
3. What is the mean score of sexual function and its domains among postmenopausal women?
4. What is the relationship between the use of CAM and sexual function, as well as its domains, among postmenopausal women?
5. What are the demographic and clinical characteristics of postmenopausal women who use CAM compared to nonusers?
6. What is the relationship between demographic and clinical information and the use of CAM among postmenopausal women?

## 3 MATERIALS AND METHODS

### 3.1 Study type and setting

This was a cross‐sectional descriptive‐correlational study. Postmenopausal women who were referred to health centers in Kerman were studied from 2020 to 2021. The city of Kerman is the capital of Kerman province, which is located in the southeast of Iran. Health centers are the first level of providing health services to the people^26^ and the city of Kerman has 47 health centers. Of these 47 health centers, 31 collaborated with researchers.

### 3.2 Sample size and sampling

The sample size was estimated at 267 using Cochran's formula for an infinite population in accordance with the study's primary objective (Z = 1.96, d = 0.06). The convenience sampling method was used. Inclusion criteria included the age range of 50–60 years old, the spouse being alive, having mental health (no history of mental disorders, bipolar, depression, postpartum depression), and at least 12 months passed since the last menstrual period.^14^, ^27^ Exclusion criteria included having a history of hysterectomy or surgery that led to abnormal menopause.

In this Project, data were collected through both online sampling form and face‐to‐face form. In the face‐to‐face form, a total of 220 questionnaires were given out to postmenopausal women referring to Kerman health centers, of which 189 were returned (a response rate of 85.9%), and 36 were excluded due to not being eligible. In the online sampling form, of 450 postmenopausal women who were contacted, 191 questionnaires were completed (a response rate of 12.44%), and 47 questionnaires were excluded for not having the inclusion criteria. All in all, a total of 297 samples were included in the study. The total response rate was 56.71%.

### 3.3 Measures

The measures were demographic characteristics and background form, Complementary and Alternative Medicine Questionnaire (CAMQ), Female Sexual Function Index (FSFI).

### 3.4 Demographic characteristics and background form

This form included the participant and her spouse's age, length of marriage, menopause age, level of education, number of children, occupation, and history of chronic diseases.

### 3.5 CAMQ

A modified version of Dehghan et al.'s questionnaire was used to assess the use of CAM in postmenopausal women. This questionnaire includes 10 questions about the use of CAM (herbal medicine, dry and wet cupping, massage, acupuncture, homeopathy, soothing methods such as yoga and prayer). The answer to each question is in the form of yes or no, and if the answer is yes, the frequency of consumption (from rarely to once a day) is asked. Participants can choose the reasons for using any type of CAM, such as reducing vasomotor symptoms (hot flashes and night sweats), improving physiological and physical function, reducing sleep disorders, improving sexual function and urogenital disorders, improving menopausal mood swings, reducing hormone therapy complications, and improving the quality of life. There is another question about consulting a doctor about using CAM that can be answered with yes or no.

Also, there were nine questions to measure satisfaction with using complementary medicine, including accessibility, ease of use, harmlessness, noninterference with daily activities, worries about interaction with other treatments, feeling good after using the treatment, suggestion of this method to others, and cost‐effectiveness. This part is scored on a five‐point Likert scale (4 = very satisfied, 3 = satisfied, 2 = dissatisfied, 1 = very dissatisfied, and 0 = no comment). The satisfaction score varied from 0 to 36. The higher the score, the greater the satisfaction. The content validity index of the questionnaire was confirmed by 10 faculty members trained in CAM. Also, its reliability was determined by a pilot study on 30 people of the target population who used CAM. The Cronbach's *α* was reported as 0.77.^28^ In another study, Dehghan et al. did some minor revisions on the questionnaire; They reported the content validity index of the questionnaire as 0.96, and for the internal consistency Cronbach's *α* coefficient was 0.85.^25^ In the present study, the Cronbach's *α* coefficient was obtained 0.74.

### 3.6 FSFI

Rosen et al.'s FSFI was used to assess sexual performance in postmenopausal women.^29^ This index includes 19 questions which measure women's sexual function in six domains of desire (two questions), arousal (four questions), lubrication (four questions), orgasm (three questions), satisfaction (three questions), and pain (three questions) during the last 4 weeks.^26^ The score ranges for each domain are 0–5 or 1–5. A score of zero indicates that the person has not had sexual activity during the last 4 weeks.^29^ Since the number of questions and score range of each domain is different, to balance the domains with each other, the scores obtained from the questions of each domain are added together and then multiplied by a factor for each domain: desire (0.6), arousal (0.3), lubrication (0.3), orgasm (0.4), satisfaction (0.4), and pain (0.4). Therefore, for each domain, the minimum and maximum scores are desire (1.2–6), arousal (0–6), lubrication(0–6), orgasm (0–6), satisfaction (0.8–6), and pain (0–6). By adding the scores of the six domains together, the total score of the index is obtained. The minimum and maximum total scores are 2 and 36. A higher score indicates better sexual function. The cut‐off point for the total score is 28 and for the domains are: desire 3.3, arousal 3.4, lubrication 3.4, orgasm 3.4, satisfaction 3.8, and pain 3.8. In other words, scores higher than the cut‐off point indicate good sexual performance and the total score <28 indicates sexual dysfunction.^29^ The validity of this questionnaire was confirmed by Rosen et al. with a Cronbach's *α* coefficient of 0.89.^29^ In Iran, the validity and reliability of the Persian version of this questionnaire have been reported by Mohammadi et al. with a Cronbach's *α* coefficient of 0.70.^30^ In the present study, the Cronbach's *α* coefficient was obtained 0.94.

### 3.7 Data collection

Due to the prevalence of corona, this study employed a mixed‐methods approach. Sampling was done in both face‐to‐face and online forms in an 8‐month period from January until August 2021. All participants were informed of the study goals and their voluntary participation. Informed consent was obtained. Sampling was done after receiving health services so that there would be no disruption in the service delivery process for these women and they would have enough time to answer the questionnaire. If the subjects were illiterate, a questionnaire was read to them. In the online form, questionnaires were prepared and given out via social networks like Telegram, Whatsapp, and SMS. The researcher, after receiving the contact numbers of postmenopausal women from health centers, identified the eligible individuals and invited them by telephone call to participate in the study. The objectives of the research, its implementation method, and how to fill out an online questionnaire were explained. If they wished to participate in the study, they were asked to sign and read the written consent form by referring to a link on the consent form. Then the link to the questionnaire was sent to eligible postmenopausal women.

### 3.8 Data analysis

IBM SPSS Statistics software version 25 was used. Descriptive statistics (frequency, percentage, mean, and standard deviation) were used to describe the demographic characteristics and background variables, the use of CAM, the cause and satisfaction of CAM, consultation with the physician, and sexual function. A *χ* ^2^ and an independent *t*‐test were used to determine the similarity between the two groups of users and nonusers of CAM in terms of demographic characteristics and background variables. In accordance with the objectives of the study, if the parametric conditions (normal distribution according to Kolmogorov–Smirnov test and equal variances) were met, parametric test (independent t to compare the mean scores of sexual function of postmenopausal women in the two groups of users and nonusers of CAM) was used, and otherwise, its nonparametric equivalent (Mann–Whitney U test) was used. A logistic regression test was also used to determine the effect of confounding variables. A significance level of lower 0.05 was considered.

## 4 RESULTS

The participants' average age was 54.99 ± 3.08 years. The majority of those who took part in the survey were educated yet unemployed. Only 4% of the individuals had no children. Nearly half of those who took part in the study had a history of chronic illness (Table 1).

**Table 1:** Comparison of demographic and clinical information in CAM users and nonusers.

		CAM user			
Variable	Mean (SD)	Yes (*n* = 196) (Mean/SD)	No (*n* = 101) (Mean/SD)	Independent *t* test	*p* Value
Age (year)	54.99 (3.08)	54.95 (3.16)	55.06 (2.93)	0.28	0.78
Spouse's age (year)	59.96 (5.94)	59.95 (5.53)	59.99 (6.70)	0.06	0.96
Duration of marriage (year)	33.32 (6.78)	34.14 (6.47)	31.72 (7.12)	−2.97	0.003
Time past from the last menstruation (year)	5.41 (3.77)	5.23 (3.65)	5.77 (3.98)	1.19	0.23

	***N* (%) (*n*** = **297)**	**Yes (*N*/%) (*n*** = **196)**	**No (*N*/%) (*n*** = **101)**	***χ* ^2^ test**	***p* Value**
Education level					
Uneducated	26 (8.8)	18 (69.2)	8 (30.8)	1.81	0.61
Middle/high school	68 (22.9)	49 (72.1)	19 (27.9)		
Diploma	106 (35.7)	68 (64.2)	38 (35.8)		
Academic education	97 (32.6)	61 (62.9)	36 (37.1)		
Children No.					
0	12 (4.0)	8 (66.7)	4 (33.3)	14.26	0.01
1	27 (9.1)	11 (40.7)	16 (59.3)		
2	73 (24.6)	51 (69.9)	22 (30.1)		
3	85 (28.6)	51 (60.0)	34 (40.0)		
4	54 (18.2)	43 (79.6)	11 (20.4)		
≥5	46 (15.5)	32 (69.6)	14 (30.4)		
Job					
Unemployed/housewife	210 (70.7)	136 (64.8)	74 (35.2)	0.63	0.73
Employed	36 (12.1)	24 (66.7)	12 (33.3)		
Retired	51 (17.2)	36 (70.6)	15 (29.4)		
History of diabetes					
Yes	33 (11.1)	27 (81.8)	6 (18.2)	4.14	0.04
No	264 (88.9)	169 (64.0)	95 (36.0)		
History of hypertension					
Yes	71 (23.9)	51 (71.8)	20 (28.2)	1.42	0.23
No	226 (76.1)	145 (64.2)	81 (35.8)		
History of other chronic disease					
Yes	144 (48.5)	73 (50.7)	71 (49.3)	29.15	<0.001
No	153 (51.5)	123 (80.4)	30 (19.6)		

Abbreviations: CAM, complementary and alternative medicine; SD, standard deviation.

Overall, 66% of participants (*n* = 196, 95% confidence interval = 60.6–71.4) said they had used at least one type of CAM in the preceding year. Furthermore, in the previous year, 23.6% of participants used only one type of CAM, 22.9% used two types of CAMs, 12.1% used three types of CAMs, 4.4% used four, and 3% used five or six types of CAMs. A total of 50.5% of participants had used prayer, 30.6% herbal medicine, 18.9% relaxation and meditation, 11.4% nutritional supplements, 9.8% dry cupping, 8.4% wet cupping, and 1% acupressure, 0.7% acupuncture, and 0.3% homeopathy (Table 2). Table 2 lists the most prevalent reasons for using CAM.

**Table 2:** The use of CAMs and the reasons for using each type of CAMs in menopause women.

			Reasons for using the CAM methods (*n* [%]^a^)							
Variable	Frequency of the users (%)	Confidence interval of percentage (%)	Reducing vasomotor symptom	Improving physical function	Reducing sleep disorders	Improving sexual function	Improving mood status	Reducing complication of hormonotherapy	Improving QOL	Others
Medicinal herbs	91 (30.6)	25.9–36.0	43 (14.5)	68 (22.9)	65 (21.9)	36 (12.1)	46 (15.5)	11 (3.7)	74 (24.9)	14 (4.7)
Dry cupping	29 (9.8)	6.4–13.1	17 (5.7)	25 (8.4)	15 (5.1)	14 (4.7)	18 (6.1)	7 (2.4)	23 (7.7)	7 (2.4)
Wet cupping	25 (8.4)	5.7–11.8	19 (6.4)	19 (6.4)	14 (4.7)	14 (4.7)	13 (4.4)	9 (3.0)	21 (7.1)	9 (3.0)
Massage	23 (7.7)	4.7–10.8	8 (2.7)	16 (5.4)	15 (5.1)	11 (3.7)	10 (3.4)	7 (2.4)	17 (5.7)	9 (3.0)
Nutritional supplements	34 (11.4)	8.1–15.5	22 (9.8)	19 (6.4)	19 (6.4)	19 (6.4)	20 (6.7)	15 (5.1)	28 (9.4)	19 (6.4)
Acupressure	3 (1.0)	0.6–2.4	–	–	1 (0.3)	–	1 (0.3)	–	1 (0.3)	–
Acupuncture	2 (0.7)	0.0–1.7	2 (0.7)	2 (0.7)	2 (0.7)	2 (0.7)	2 (0.7)	2 (0.7)	2 (0.7)	–
Homeopathy	1 (0.3)	0.0–1.0	1 (0.3)	1 (0.3)	1 (0.3)	1 (0.3)	1 (0.3)	1 (0.3)	1 (0.3)	–
Relaxation and meditation	56 (18.9)	14.5–23.6	36 (12.1)	46 (15.5)	41 (13.8)	32 (10.8)	39 (13.1)	9 (3.0)	52 (17.5)	9 (3.0)
Prayer	150 (50.5)	45.1–56.2	34 (11.4)	41 (13.8)	57 (19.2)	30 (10.1)	71 (26.9)	30 (10.1)	132 (44.4)	37 (12.5)

Abbreviations: CAM, complementary and alternative medicine; QOL, quality of life.

^a^ Each individual could select more than one reason for using each type of COMs.

Before taking nutritional supplements, 94.11% of the participants sought medical counsel. Before utilizing dry and wet cupping, 72.41% and 64% of the participants sought medical advice, respectively. Before employing massage, 43.48% of the people sought medical counsel. Before using herbal medicine, 36.26% of the people sought medical counsel. The mean satisfaction score for CAM use was 21.7 ± 5.84 (Min = 0 and Max = 36), which was higher than the scale's midpoint of 18.

The mean score of sexual function was 17.96 ± 7.50 (Table 3). Only 1.3% of participants had good sexual function. According to different domains of sexual function, 52.9% had good desire, 49.5% good arousal, 34.7% good lubrication, 56.9% good orgasm, and 2% had no pain, and 52.5 were satisfied.

**Table 3:** Comparison of sexual function in CAM users and nonusers.

		CAM user			
Variable	Mean (SD)	Yes (*n* = 196) (Mean/SD)	No (*n* = 101) (Mean/SD)	Statistical test	*p* Value
Desire	3.25 (1.36)	3.23 (1.38)	3.29 (1.34)	t = 0.37	0.71
Arousal	3.29 (1.93)	3.18 (1.85)	3.50 (2.06)	Z = −1.41	0.16
Lubrication	2.61 (1.28)	2.65 (1.26)	2.52 (1.33)	t = −0.80	0.42
Orgasm	3.06 (1.53)	3.07 (1.46)	3.06 (1.65)	Z = −1.29	0.20
Satisfaction	4.08 (1.70)	3.98 (1.60)	4.28 (1.89)	Z = −1.86	0.06
Pain	1.66 (1.12)	1.72 (1.15)	1.56 (1.05)	t = 0.11	0.30
Total score of sexual function	17.96 (7.50)	17.82 (7.27)	18.21 (7.94)	Z = −1.10	0.27

Abbreviations: CAM, complementary and alternative medicine; SD, standard deviation.

There were no significant differences between CAM users and nonusers regarding sexual functions and all its domains (Table 3). Among the study variables, only the duration of marriage, number of children, history of diabetes, and history of other chronic diseases were associated with CAM use (Table 1). For further analysis, these variables were included in a logistic regression model. The results showed that participants with no history of other chronic diseases used CAM 3.53 times (95% confidence interval for odds ratio: 2.01–6.20) more than participants with a history of other chronic diseases. In addition, the other variables were no longer associated with CAM use.

## 5 DISCUSSION

The aim of this study was to investigate the use of CAM and its relationship with sexual function among postmenopausal women referring to health centers in Kerman.

The results of the present study showed that more than half of menopausal women used at least one type of CAM in the last year. About half of the participants used prayer, almost a third herbal medicine, less than a quarter relaxation and meditation, and the least used methods were wet cupping, acupressure, acupuncture, and homeopathy, respectively. Arentz et al. in Australia showed that more than almost three quarters of women with polycystic ovary syndrome reported using complementary medicines, usually dietary supplements and herbal supplements.^31^ Witteman et al. found that more than three quarters of women with UTI used complementary or self‐care strategies in addition to standard treatment, and almost more than half used CAM/self‐care strategies to manage vaginal symptoms. Cranberries (51.9%), vitamin C (43.8%), and d‐mannose (32.7%) were the most often reported UTI‐related dietary supplements.^32^ The types and patterns of CAM use are socioculturally determined and are generally influenced by society, history, and occasionally religion.^25^ This study was different from our study in terms of samples, measures, and cultural factors. In Iran, the majority of people are Muslims, and religious practices such as praying and meditating are the daily practices of the Muslim people of Iran.^25^ Also, due to the historical background of the use of complementary medicine, the use of complementary medicine is common in Iran.

The results of the present study showed that the sexual function of menopausal women was lower than the index's midpoint which was unfavorable. Yağmur et al. in Turkey revealed that the prevalence of sexual disorders was high among menopausal women, and almost more than half of their participants were below the cutpoint score and unfavorable.^33^ Pérez‐Herrezuelo et al. in Spain,^34^ and in Iran, Safaei et al.^32^ and Jamili et al.^35^ showed that almost three quarters, almost three quarters, and more than three quarters of menopausal women had sexual dysfunction, respectively. However, Khalesi et al. in Iran, showed that almost more than a third of their participants had female sexual dysfunction.^36^ Sexual health is influenced by personal factors, interpersonal relationships, social and family traditions, culture, and religion.^37^ Therefore, the reasons for the difference between this study and the present study are differences in personal factors, interpersonal relationships, and social and family traditions. During both menopause and premenopause, women experience several problems with their sexual life due to endocrinological and physiological changes, such as decreased sex hormones and urogenital atrophy.^38^

According to the present results, considering different domains of sexual function, about half of participants had good desire, about half had good arousal, almost more than a third had good lubrication, almost more than a half had good orgasm, nearly zero percent had no pain, and about half were satisfied. Also, the highest mean score belonged to the dimension of ssatisfaction, which was unfavorable, and the lowest mean score belonged to the dimension of pain, which was almost favorable. In the studies of Safaei et al. in Iran and Herrezuelo et al. in Spain, the highest mean score was for the dimension of satisfaction, and the lowest mean score was for sexual desire and arousal.^32^, ^34^ Also, in the study of Safaei et al., almost all samples had unfavorable desires, less than a third had favorable arousal, 7% had favorable lubrication, almost the majority of the samples had unfavorable orgasm, and less than a quarter were satisfied. In the study of Jamili et al., the domains of desire and satisfaction had the highest and lowest mean scores, respectively.^35^ In Turkey, Yağmur et al. reported the highest mean score for the dimension of pain, and the lowest mean score for the dimension of desire^33^ which were inconsistent with our study. According to the result of a review article, age, estrogen deficiency, menopause pattern, chronic medical condition, sexual partner's problems, the severity of menopausal symptoms, history of obstructed labor, and medical condition are physical factors affecting the sexual function of postmenopausal women.^39^ Differences in samples' age and the culture of samples from the point of view of being taboo in reporting sexual issues and problems can be the reasons for the inconsistency of studies with the results of the present study.

In the present study, the duration of marriage, number of children, history of diabetes, and history of other chronic diseases were associated with CAM use. In the study of Movahed Majd et al., results showed that variables such as occupational status, attitudes toward traditional and complementary medicine, social networks, and accessibility to traditional and complementary medicine were positively and significantly correlated with the use of traditional and complementary medicine,^40^ which was inconsistent with the results of the present study.

The results of the present study showed that there were no significant differences between CAM users and nonusers regarding sexual function and all its domains, so that the use of complementary medicine did not improve the sexual function of menopausal women. However, Johnson et al. showed that mind and body practices, including hypnosis and cognitive behavioral therapy, could be effective in the treatment of some common and problematic symptoms of menopause (vasomotor sexual function, some sleep regulations). Other mental and physical practices (biofeedback, mindfulness‐based stress reduction, relaxation techniques) may reduce stress and improve quality of life in menopausal women, but have not been proven to be effective for specific symptoms of menopause.^20^ The result of a meta‐analysis showed that aromatherapy with Neroli or Lavender Oil (Monopreparation) and aromatherapy with oil in combination with Lavender, Fennel, Geranium, and Rose significantly improved human sexual function.^41^ In Oaklery et al.'s study, sexual function, particularly desire, arousal, lubrication, and orgasm, improved after acupuncture therapy.^42^ The results of Esposito et al.'s study showed that the Mediterranean diet improved female sexual function in women with metabolic syndrome and reduced C‐reative protein levels. However, no single sexual domain (desire, arousal, lubrication, orgasm, satisfaction, or pain) was significantly improved by food treatment, implying that lifestyle modifications may help the entire female libido.^21^ As well, the results of another systematic review showed that acupuncture alters sex hormones in various gynecological conditions in women.^22^ The result of Taavoni et al.'s indicated that Aphrodite (an herbal supplement made from a combination of several plants such as ginger, saffron, cinnamon, and Kharkhask) use can improve orgasm and sexual desire in postmenopausal women.^23^ According to Abedi et al.'s study results, fennel vaginal cream is an effective way to improve sexual performance in postmenopausal women. This product is recommended for use in women with sexual dysfunction who have contraindications to hormone therapy.^18^ The results of the mentioned studies were inconsistent with the results of the present study. Perhaps the differences in health systems in the use of complementary medicine, the participants' level of knowledge, and the prevalence and acceptance of a particular type of CAM in different communities could be the causes of this contradiction.

## 6 STUDY LIMITATION

Sexual issues are considered taboo in many social and cultural contexts. In Iran, sexual problems and issues are considered taboo, too. In this approach, sexual dysfunction may be perceived and reported as less real in this context. We used female researchers to gather information to overcome this limitation. The COVID‐19 virus outbreak has also decreased the number of postmenopausal women visiting health centers. To overcome this limitation, convenience sampling was used in conjunction with a mix of face‐to‐face and virtual contact. Lack of knowledge about some types of CAMs in postmenopausal women was also one of the limitations of this study. Another limitation was the selection of menopausal women from an urban area. Conducting national and international studies makes it possible to generalize these results. The most important factor related to sexual dysfunction is vaginal atrophy and because hormone replacement therapy in Iran is not common, it is anticipated that most women sought CAM therapy for their vaginal atrophy, that was not evaluated by authors and it is an instrument limitation of this study.

## 7 CONCLUSION

According to the result, the sexual function of postmenopausal women was not at an acceptable level. In addition, while the use of CAM was high among postmenopausal women, there were no significant differences between CAM users and nonusers about sexual functions and all its domains. Sexual dysfunction in postmenopausal women is common, therefore, education to maintain and improve the general sexual health of postmenopausal women is important. However, there were no significant association between using CAMs and sexual functions, further studies are needed to examine more closely the relationship between the other CAMs usage and sexual function.

## AUTHOR CONTRIBUTIONS

**Mahlagha Dehghan**: Conceptualization; data curation; formal analysis; methodology; project administration; software; supervision; writing—review and editing. **Zahra Isari**: Data curation; supervision; writing—original draft. **Mohammad Hossein Abbaszadeh**: Conceptualization; data curation; writing—original draft. **Seyed Amirreza Shafiee Babaei**: Conceptualization; writing—original draft. **Zahra Kamali Narab**: Data curation. **Alireza Malakoutikhah**: Writing—original draft. **Asma Ghonchehpour**: Data curation; methodology; supervision; writing—original draft; writing—review and editing.

## CONFLICT OF INTEREST STATEMENT

The authors declare no conflict of interest.

## ETHICS STATEMENT

The ethical committee of Kerman University of Medical Sciences approved the study (IR. KMU. REC.1399.444). After approval, permission was issued to the management of the Health Care Centers. Some oral information, including the goals and objectives of the study, the confidentiality and anonymity of the data, and that the participants were free to withdraw from the study at any time were provided by the researchers. Then written informed consent was taken individually. All methods were carried out in accordance with relevant guidelines and regulations and Declaration of Helsinki.

## TRANSPARENCY STATEMENT

The lead author Asma Ghonchehpour affirms that this manuscript is an honest, accurate, and transparent account of the study being reported; that no important aspects of the study have been omitted; and that any discrepancies from the study as planned (and, if relevant, registered) have been explained.

## Data Availability

The data sets used and/or analyzed during the current study are available from the corresponding author on reasonable request.
